# Supplementary material for: Determinants of a mobile phone-based Interactive Voice Response (mIVR) system for monitoring childhood illnesses in a rural district of Ghana: Empirical evidence from the UTAUT model
Source: PLoS One. 2021 Mar 11;16(3):e0248363. doi: 10.1371/journal.pone.0248363 (PMC7951827; doi:10.1371/journal.pone.0248363)
Supplement: S1 Fig — (DOCX) [file pone.0248363.s001.docx]

**S1 Fig: Caregivers reasons for non-use of mIVR system**
