# Supplementary material for: Determinants of a mobile phone-based Interactive Voice Response (mIVR) system for monitoring childhood illnesses in a rural district of Ghana: Empirical evidence from the UTAUT model
Source: PLoS One. 2021 Mar 11;16(3):e0248363. doi: 10.1371/journal.pone.0248363 (PMC7951827; doi:10.1371/journal.pone.0248363)
Supplement: S1 Table — (DOCX) [file pone.0248363.s002.docx]

**S1 Table. Analysis of mobile phone ownership among caregivers**

| **Variables** | **Number of respondents (n=304)** | **% of respondents with mobile phone** | **P- value** |
| --- | --- | --- | --- |
| **Age (years)** |  |  | 0.001 |
| ≤ 20 | 16 | 59.26 |  |
| 21-30 | 155 | 87.08 |  |
| 31-40 | 105 | 86.78 |  |
| 41-50 | 23 | 100.0 |  |
| > 50 | 5 | 100.0 |  |
| **Educational level** |  |  | 0.080 |
| No education | 67 | 87.01 |  |
| Primary | 71 | 81.61 |  |
| Middle/JHS | 99 | 82.50 |  |
| Secondary/Vocational | 54 | 94.74 |  |
| Tertiary | 13 | 100.0 |  |
| **Wealth quintiles (SES)** |  |  | 0.001 |
| 1^st^ quintile (poorest) | 43 | 60.56 |  |
| 2^nd^ quintile | 63 | 88.73 |  |
| 3^rd^ quintile | 78 | 93.98 |  |
| 4^th^ quintile | 56 | 91.80 |  |
| 5^th^ quintile (wealthiest) | 64 | 94.12 |  |
| **Phone Experience** |  |  | 0.300 |
| Experience | 171 | 84.24 |  |
| Inexperience | 133 | 88.08 |  |
| **Gender** |  |  | 0.003 |
| Male | 48 | 100.0 |  |
| Female | 256 | 83.66` |  |

JHS, Junior High School; SES, Socio-economic Status
