# Supplementary material for: Determinants of a mobile phone-based Interactive Voice Response (mIVR) system for monitoring childhood illnesses in a rural district of Ghana: Empirical evidence from the UTAUT model
Source: PLoS One. 2021 Mar 11;16(3):e0248363. doi: 10.1371/journal.pone.0248363 (PMC7951827; doi:10.1371/journal.pone.0248363)
Supplement: S2 Table — (DOCX) [file pone.0248363.s003.docx]

**S2 Table. Distribution of use of the IVR system by caregivers**

| **Variable** | **Frequency** | **Percentage (%)** |
| --- | --- | --- |
| **Ever used IVR system** **(N=354)** |  |  |
| Yes | 101 | 28.53 |
| No | 253 | 71.47 |
|  |  |  |
| **Intension to use in the future** **(N=354)** | N=354 |  |
| Yes | 328 | 92.66 |
| No | 1 | 0.28 |
| Not sure | 25 | 7.06 |
|  |  |  |
| **Reasons for use (n=101)** | N=101 |  |
| Seeking care for my child health | 91 | 59.48 |
| It’s time saving | 6 | 3.92 |
| Enhanced access to health care and information | 9 | 5.88 |
| Cost reduction | 9 | 5.88 |
| Opportunity to talk to doctor | 38 | 24.84 |
|  |  |  |
| **Frequency of Use of IVR system** **(n=101)** | N=101 |  |
| 1 | 53 | 52.48 |
| 2 | 39 | 38.61 |
| 3 | 7 | 6.93 |
| 4 | 2 | 1.98 |

IVR, Interactive Voice Response
