# Supplementary material for: Determinants of a mobile phone-based Interactive Voice Response (mIVR) system for monitoring childhood illnesses in a rural district of Ghana: Empirical evidence from the UTAUT model
Source: PLoS One. 2021 Mar 11;16(3):e0248363. doi: 10.1371/journal.pone.0248363 (PMC7951827; doi:10.1371/journal.pone.0248363)
Supplement: Attachment — Submitted filename: Response to Reviewers.docx [file pone.0248363.s004.docx]

Dear Editor,

Thank you very much for the comments raised by the reviewers following the review of the manuscript submitted for consideration (coded PONE-D-20-32440) for publication in your reputable journal.

I hereby write to address the comments from the reviewers:

Question 3:  Have the authors made all data underlying the findings in their manuscript fully available?

**Reviewer #1**: No

**Response**: The data underlying the findings in the manuscript have been deposited in public repository. This is accessible through this URL link (<https://doi.org/10.3886/E124361V1>) which was provided in the submission of the manuscript. All files are available from the Inter-university Consortium for Political and Social Research (ICPSR) database.

**Reviewer’s Comment**: In the study design area section, it is stated that the study was carried out in 7 randomly selected communities out of 115 communities: (a) I think the authors should state why they chose 7 out of 115 and not more. (b) The authors should also show the number of participants in each of the 7 communities in which they conducted their study.

**Response**:

(a) The study area, Asante Akim North District has been profiled into 23 Enumeration Areas (EAs) by the district. Out of these, 7 were selected for this study. The 7 EAs were labeled as communities instead of enumeration areas in the manuscript. Each EA has an average of 5 smaller communities. This change has therefore been effected in the main manuscript. The selection of 7 EAs was also because certain areas had network challenges.

(b) The various number of study participants in each enumeration area (EA) is illustrated in Table 2: Socio-demographic profile of participants.

Thank you very much.

Yours faithfully,

Timothy Kwabena Adjei

(Corresponding author)
